# Near-channel classifier: symbiotic communication and classification in high-dimensional space

**DOI:** 10.1186/s40708-021-00138-0

**Published:** 2021-08-17

**Authors:** Michael Hersche, Stefan Lippuner, Matthias Korb, Luca Benini, Abbas Rahimi

**Affiliations:** 1 Integrated Systems Laboratory, ETH Zurich, Zurich, Switzerland; 2 IBM Research-Zurich, Zurich, Switzerland; 3 Institute of Microelectronics and Integrated Circuits, Bundeswehr University, Munich, Germany; 4 Department of Electrical, Electronic, and Information Engineering, University of Bologna, Bologna, Italy

**Keywords:** High-dimensional computing, Communication, Classification, Electromyography

## Abstract

Brain-inspired high-dimensional (HD) computing represents and manipulates data using very long, random vectors with dimensionality in the thousands. This representation provides great robustness for various classification tasks where classifiers operate at low signal-to-noise ratio (SNR) conditions. Similarly, hyperdimensional modulation (HDM) leverages the robustness of complex-valued HD representations to reliably transmit information over a wireless channel, achieving a similar SNR gain compared to state-of-the-art codes. Here, we first propose methods to improve HDM in two ways: (1) reducing the complexity of encoding and decoding operations by generating, manipulating, and transmitting bipolar or integer vectors instead of complex vectors; (2) increasing the SNR gain by 0.2 dB using a new soft-feedback decoder; it can also increase the additive superposition capacity of HD vectors up to 1.7$\times$ in noise-free cases. Secondly, we propose to combine encoding/decoding aspects of communication with classification into a single framework by relying on multifaceted HD representations. This leads to a near-channel classification (NCC) approach that avoids transformations between different representations and the overhead of multiple layers of encoding/decoding, hence reducing latency and complexity of a wireless smart distributed system while providing robustness against noise and interference from other nodes. We provide a use-case for wearable hand gesture recognition with 5 classes from 64 EMG sensors, where the encoded vectors are transmitted to a remote node for either performing NCC, or reconstruction of the encoded data. In NCC mode, the original classification accuracy of 94% is maintained, even in the channel at SNR of 0 dB, by transmitting 10,000-bit vectors. We remove the redundancy by reducing the vector dimensionality to 2048-bit that still exhibits a graceful degradation: less than 6% accuracy loss is occurred in the channel at − 5 dB, and with the interference from 6 nodes that simultaneously transmit their encoded vectors. In the reconstruction mode, it improves the mean-squared error by up to 20 dB, compared to standard decoding, when transmitting 2048-dimensional vectors.

## Introduction

**Fig. 1:**
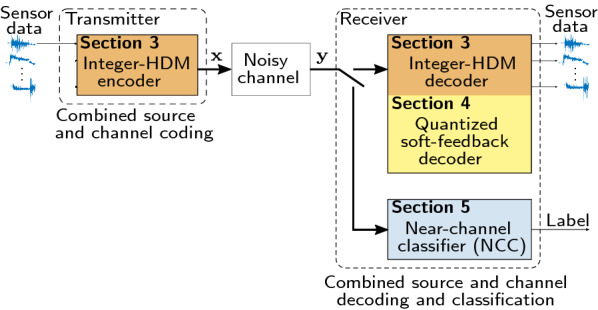
Overview of this work with colors pointing to Sects. 3, 4 and 5 in the paper. Sensor data are encoded to high-dimensional vector ${\mathbf {x}} \in {\mathbb {Z}}^ D$ using a novel Integer-HDM encoder and transmitted over a noisy channel. At the receiver, the perturbed vector ${\mathbf {y}}$ is either decomposed by an optimized soft-feedback decoder (that improved Integer-HDM decoder) to reconstruct the sensory data, or directly classified by a near-channel classifier (NCC) without any decoding step

With the rapid growth in the number of deployed sensing nodes in the physical world [1–3] and their interconnection with sensor networks, Swarms, or the Internet of Things [4], the world around us has become noticeably smarter [5]. Machine learning (ML), either being deployed in the cloud or at the edge near the sensor [6–9], plays a crucial role in extracting relevant information from the sensors and data spread in space. The standard approach is to create a *layered* system that separates the communication, including source and channel coding, from the ML. Such a layered approach imposes unnecessary transitions between the layers which adds to latency and complexity. Hence, there is a need for a representational system that effectively merges communication and ML layers into a single framework for wireless distributed smart sensing systems, as shown in Fig. 1.

One viable option is to exploit novel representations in high-dimensional (HD) computing [10–13], where data are represented by very long, random vectors (dimension $D=1000$ – 10,000). Inspired by the size of the brain’s circuits, these vectors are *holographic* and (pseudo)random with independent and identically distributed (i.i.d.) components [10]. As the vectors are composed through a set of well-defined mathematical operations, they can be queried, decomposed [14], and reasoned about [15, 16]. For learning and classification tasks, HD computing was initially applied to text analytics, where each discrete symbol can be readily mapped to a random vector to be combined across text [17–20]. More recently, HD computing has been extended to operate with a set of analog inputs [21–25], mainly in several biosignal processing applications, or with event-driven inputs from neuromorphic dynamic vision sensors [26].

HD vectors are very tolerant to noise, variations, or faulty computations due to their redundant i.i.d. representation, in which information symbols are spread holographically across many components [10, 20, 27]. This makes HD computing a prime candidate for implementation on emerging nanoscale hardware operating at low signal-to-noise (SNR) conditions [28–30]. In a similar vein, methods have been proposed to make use of the robustness of HD vectors in various communication layers [31–37]. Particularly, recent hyperdimensional modulation (HDM) [33] can be interpreted as a spreading modulation scheme whose spreading gain linearly improves with the vector dimension, allowing higher error tolerance with increased dimensionality. Multiple spread vectors are *superposed* before transmission; at the receiver, an iterative feedback decoder denoises the query vector by subtracting the estimated vectors. In low SNR channels where each value cannot be reliably demodulated, HDM can still achieve successful demodulation of symbols without requiring an explicit error correction.

In an initial effort, it was shown that HDM exhibits a comparable bit error rate (BER) to that of low-density parity check (LDPC) and Polar codes at a lower number of operations in decoding [33]. Moreover, HDM was shown to be more collision tolerant than conventional orthogonal modulations (e.g., OFDM) in highly congested low power wide area networks [34]. However, the HDM proposed in [33] represents symbols using complex-valued components in a vector, hence we call it Complex-HDM, which requires more bits per symbol to be transmitted and involves energy-hungry fast Fourier transform (FFT) operations in encoding and decoding.

Here, we first address these shortcomings of Complex-HDM by simplifying its encoding/decoding operations, and improving its SNR gain. Next, we demonstrate how our approach can effectively blur the boundaries between communication and ML by relying on a unified HD representation system. This paper makes the following three main contributions (highlighted in Fig. 1 as well).

First, in Sect.  3, we propose Integer-HDM that superposes bipolar vectors. These vectors can be rematerialized in an encoder with a combination of simple lookup and permutation operations that are hardware-friendly [38]. Further, the burden of decoding complexity is lowered by using associative memory (AM) searches, purely with integer arithmetic instead of performing FFT. Such best match searches use cheap clean-up operations, which scale better than FFT searches on long codes, and can be efficiently implemented with analog in-memory computing [30]. Our Integer-HDM achieves the same SNR gain as the Complex-HDM [33] under additive Gaussian white noise (AWGN) without relying on the expensive FFT operations in encoding and decoding.

Secondly, to improve the SNR gain, we propose a soft-feedback decoding mechanism which additionally takes the estimation’s confidence into account (Sect.  4). Although the soft-feedback involves floating-point operations, it improves the SNR gain of the Integer-HDM by 0.2 dB at a BER of 10$^{-4}$. To simplify the soft-feedback decoder, it is quantized to 4.1 fixed-point without any degradation in the SNR gain under AWGN. Further, we have observed that our soft-feedback decoder can be combined with an optimized minimum mean-squared error (MMSE) readout to increase the number of superposed vectors, which can be successfully decomposed in a noise-free case. This effectively improves the capacity of HD superposition by $1.7\times$ for noise-free information retrieval; we improve the number of encoded information bits in a 500-dimensional HD vector [14] from 0.7 to 1.2 bits/dimension.

Thirdly, we propose to combine channel coding, source coding, and ML classification into a single unified layer exploiting multifaceted HD representations. This approach avoids transformations between representations and the addition of multiple layers of encoding/decoding. The approach is inspired by the structural similarities between the Integer-HDM encoding and the spatial feature encoding in HD classifiers used for multichannel biosignal classification tasks [22, 25]. In practice, we reuse the spatial encoding for both data transmission and classification; hence, we avoid the transition between different representations. The encoded vector can be reliably transmitted to the receiver, where it is either decoded to analyze the underlying data, or directly classified, enabling near-channel classification (NCC). In Sect. 5, we present a use case for wearable hand gesture recognition (5-class) based on electromyography (EMG) signals from 64 sensors [22] where encoded vectors are transmitted to perform either NCC, or reconstruct the underlying features at the receiver. In NCC mode, the 10,000-bit representation shows great robustness by maintaining the noise-free accuracy of 94% at SNR as low as 0 dB. Reducing the vector dimension to 2048-bit—where there is no redundancy—also exhibits graceful degradation in the presence of AWGN and interference from other sensor nodes, allowing up to $-5$ dB SNR and up to 6 simultaneously sending sensor nodes at less than 6% accuracy loss, compared to the noise-free case. Moreover, the soft-feedback decoder guarantees successful reconstruction of the features even in noisy environments and improves the mean-squared reconstruction error by up to 20 dB compared to standard decoding at dimension $D=2048$.

In the remainder of the paper, Sect. 2 provides background into HD computing, the creation and decomposition of HD superpositions, and HDM. Section 6 concludes the paper.

## Background

### High-dimensional computing

The brain’s circuits are massive in terms of numbers of neurons and synapses, suggesting that large circuits are fundamental to the brain’s functioning. HD computing [10]—aka holographic reduced representations [12], semantic pointer architecture [39], or vector symbolic architectures [13, 40]—explores this idea by looking at computing with vectors as ultrawide words. These vectors are *D*-dimensional (the number of dimensions is in the thousands) and (pseudo)random with independent and identically distributed (i.i.d.) components. They thus conform to a holographic or holistic representation: the encoded information is distributed equally over all the *D* components such that no component is more responsible for storing any piece of information than another. Such representation maximizes robustness for the most efficient use of redundancy [10].

In this work, we focus on multiply–add–permute (MAP) architectures [13], which define the multiplication ($*$) as the element-wise multiplication between two vectors, the addition ($+$) as the element-wise addition among multiple vectors, and the permutation ($\Pi$) as the random shuffling of the vector elements. Multiplication and permutation yield dissimilar vectors compared to their input vector, whereas addition preserves similarity and is often used to represent sets. The permutation can be realized with hardware-friendly, cyclic shifts ($\rho$). We compare two *D*-dimensional vectors ${\mathbf {x}}$ and ${\mathbf {y}}$ with the cosine similarity:

1 $$\begin{aligned} c = \frac{<{\mathbf {x}},{\mathbf {y}}>}{||{\mathbf {x}}||_2 \cdot ||{\mathbf {y}}||_2}, \end{aligned}$$

where $<.,.>$ is the $\ell _2$-inner product and $||.||_2$ the $\ell _2$-norm. The cosine similarity reflects the angle between vectors, neglecting their length/norm.

Creating HD representations starts with building a dictionary (aka item memory) ${\mathbf {IM}} = \lbrace {\mathbf {e}}_1, {\mathbf {e}}_2, ..., {\mathbf {e}}_N\rbrace$, where ${\mathbf {e}}_i \in \lbrace -1,1\rbrace ^D$ are atomic vectors with the elements in each vector being a Rademacher random variable (i.e., equal chance of values being “$-1$” or “$+1$”). 

The high dimensionality guarantees all elements in the dictionary to be orthogonal with high probability, aka quasi-orthogonality. Information can be encoded by HD superposition: a string of information symbols $(q_1,q_2,...,q_V),\, q_i \in \lbrace 1,2,...,N\rbrace \, \forall i$ is mapped to the corresponding element in the dictionary, permuted, and superposed via addition:

2 $$\begin{aligned} {\mathbf {x}}(q_1,q_2,...,q_V)&= \sum _{v=1}^V \Pi _v \left( {\mathbf {e}}_{q_v}\right) , \end{aligned}$$

3 $$\begin{aligned}&=\sum _{v=1}^V \Pi _v \left( {\mathbf {c}}(q_v)^T\cdot {\mathbf {E}}\right) , \end{aligned}$$

where $^T$ is the transpose, ${\mathbf {E}}:=\left( {\mathbf {e}}_1,{\mathbf {e}}_2,...,{\mathbf {e}}_N\right) \in \lbrace -1,1\rbrace ^{D \times N}$ the matrix representation of the IM containing the atomic vectors as columns, and ${\mathbf {c}}(q_v)\in \lbrace 0,1\rbrace ^N$ an all-zero vector except element $q_v$ that is one. Note that all permutations $\Pi _v$ are distinct.

The individual vectors in the superposition can be retrieved by the associative memory (AM) search:

4 $$\begin{aligned} {\hat{\mathbf {c}}}_v = \frac{1}{D} {\mathbf {E}}^T \cdot \Pi _v^{-1}\left( {\mathbf {x}}\right) , \end{aligned}$$

where ${\hat{\mathbf {c}}}_v \in {\mathbb {R}}^N$. The estimated index ${\hat{q}}_v$ is the one with the highest value in ${\hat{\mathbf {c}}}_v$:

5 $$\begin{aligned} {\hat{q}}_v = \mathop {\hbox {argmax}}\limits _{q=1,...,N} {\hat{\mathbf {c}}}_v[q]. \end{aligned}$$

Increasing the number of superposed vectors yields a higher information density; therefore, HD superposition can be used for compression. For example, it has been successfully applied for compressing model weights in deep neural networks [41]. However, the number of correct retrievals from highly compressed representations is limited by the number of superposed vectors *V*; an increasing *V* yields a lower signal-to-interference ratio (SIR) for retrieval.

The superposition ${\mathbf {x}}$ has integer-valued elements instead of bipolar elements; it can be bipolarized by setting negative elements to “$-1$” and positive to “$+1$”. If the number of superposed vectors is even, ties at zero are broken at random, or by simply adding another deterministic (random) vector to the superposition before bipolarizing (see [38]). Even though bipolarizing the superposition is common practice in HD computing, it heavily affects both the number of retrievable vectors and the noise resiliency in HD superposition.

### Hyperdimensional modulation

Hyperdimensional modulation (HDM) [33] superposes complex-valued vectors using the rows of the discrete Fourier transform (DFT) matrix as entries in the IM. The mapping is realized by transforming the sparse vector ${\mathbf {c}}_v$ with a DFT, whereas the readout matrix corresponds to the inverse DFT, which can be efficiently implemented with FFT and inverse FFT. Additional information is encoded by having multiple non-zero values in ${\mathbf {c}}_v$66, and modulating the non-zero values with phase-shift keying. Decoding is performed in multiple iterations, subtracting the last iteration’s estimation from the superposition for the next estimation. An additional cyclic redundancy check (CRC) validates the estimation’s correctness; if the CRC fails, the decoder searches through a list of most probable alternative solutions correcting single, double, or triple errors. This yields an SNR gain of 1.75 dB at a BER of $10^{-5}$. Overall, the presented decoding resulted in similar SNR gains compared to LDPC and Polar codes [33].

## Integer-HDM

**Fig. 2:**
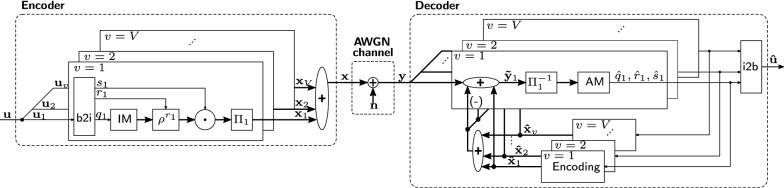
Integer-HDM encoder and decoder: binary string ${\mathbf {u}}\in \lbrace 0,1\rbrace ^k$ is encoded to HD superposition ${\mathbf {x}}\in {\mathbb {Z}}^D$ which is transmitted over an AWGN channel. The received vector ${\mathbf {y}}$ is finally decoded using an iterative unit-feedback decoder with unit feedback yielding the estimation ${\hat{\mathbf {u}}}$

This section is the first main contribution of the paper: we introduce Integer-HDM, a new modulation scheme that transmits the superposition of bipolar vectors, depicted in Fig. 2. We present a novel encoding scheme that effectively increases the IM size (i.e., the dictionary) while keeping the memory footprint small, which allows to achieve a high code throughput even on resource-limited devices. An iterative unit-feedback decoder decomposes the transmitted vector to get the estimated bit-string. Our decoder is inspired by Complex-HDM [33], but instead of requiring FFT operations it relies only on efficient AM searches. We experimentally evaluate the SNR gain in an AWGN channel and show that our novel encoding achieves the same SNR gain as Complex-HDM.

### Memory-efficient encoding

We start with the description of a memory-efficient encoding of a binary input string $\mathbf{u }$ of length *k* to a *D*-dimensional integer vector, defined as

6 $$\begin{aligned} \Phi : \quad \lbrace 0,1\rbrace ^k \xrightarrow {} {\mathbb {Z}}^D . \end{aligned}$$

We define the throughput *r* of the code in bits per channel usage

7 $$\begin{aligned} r=\frac{k}{D}. \end{aligned}$$

The ultimate goal is to find an encoding function $\Phi$ with a high code throughput while ensuring that the encoded vector is robust against errors occurring during transmission.

The left side of Fig. 2 illustrates the proposed encoding scheme. First, the input string ${\mathbf {u}}$ is divided into *V* equally sized sub-strings (${\mathbf {u}}_1,{\mathbf {u}}_2,...,{\mathbf {u}}_V$). Each sub-string is encoded separately with its corresponding encoding module. In the following we will explain the encoding of ${\mathbf {u}}_1$, and then the generalization to all other encoding modules.

First, the bit-to-index (b2i) block maps the bit-string ${\mathbf {u}}_1$ of length *k*/*V* to the IM index $q_1$, rotation index $r_1$, and sign index $s_1$. For generating the indexes, we split the bit-string into three slices that are mapped to their corresponding integer values. The resulting indexes are then further used for decoding information in the HD space.

The IM builds the central part of the encoding and serves as a random but fixed dictionary. It stores *N* bipolar vectors of dimension *D*, where the entries are drawn randomly with an equal number of “$+1$” and “$-1$”. The IM index $q_1$ is used to read out the corresponding vector in the IM. The number of information bits $k_q$ which can be encoded with an IM of size *N* is

8 $$\begin{aligned} k_q = \text {log}_2(N). \end{aligned}$$

The IM grows exponentially with the number of bits we want to encode. As a consequence, the code throughput of tightly resource-limited devices would be restricted. To relax the memory requirements, we extend the encoding by rotation encoding $\rho ^{r_1}$, which applies a cyclic rotation by $r_1$ positions to the vector. A cyclic rotation is an alternative, hardware-friendly random permutation. The shifted result is quasi-orthogonal to its input vector. The number of available shifts is limited to the number of dimensions *D*, resulting in a maximum of

9 $$\begin{aligned} k_r = \text {log}_2(D), \end{aligned}$$

additionally encoded bits. The rotation encoding *virtually increases* the IM size by factor *D*, without requiring any additional memory. In the next step, the vector is multiplied with the sign modulator $s_1\in \lbrace -1,1\rbrace$. This further gives

10 $$\begin{aligned} k_s = 1, \end{aligned}$$

bit.

We illustrate the encoding with an example assuming dimension $D=64$ and an IM size of $N=8$. The bit-string ${\mathbf {u}}_1$ contains $k_q +k_r + k_s=3+6+1=10$ bits, e.g., ${\mathbf {u}}_1=(0100100010)$. The bit-to-index block splits the bit-string into three slices (010|010001|0) and maps them to the corresponding integer indexes $q_1=2$, $r_1=17$, and $s_1=(-1)$. Finally, the encoded vector is

11 $$\begin{aligned} {\mathbf {x}}'_1 = s_1 \cdot \rho ^{r_1}\left( {\mathbf {e}}_{q_1} \right) = (-1)\cdot \rho ^{17}\left( {\mathbf {e}}_{2} \right) . \end{aligned}$$

The described encoding steps are identical among different encoding blocks; the same IM is shared among all blocks. In the last step, the encoded vectors are permuted with a unique, random permutation $\Pi _v$ per encoding block and superposed, resulting in the final vector ${\mathbf {x}}$. The final throughput of the code is

12 $$\begin{aligned} r&=\frac{V(k_q+k_r+k_s)}{D}, \end{aligned}$$

13 $$\begin{aligned}&= \frac{V\left( {\mathrm {log}}_2(N)+\text {log}_2(D)+1\right) }{D}. \end{aligned}$$

### FFT-free decoding based on associative memory

We present an iterative unit-feedback decoder, depicted in Fig. 2, which decomposes the transmitted vector ${\mathbf {y}}$ to estimate the bit-string ${\hat{\mathbf {u}}}$. It consists of an estimation and a feedback stage. In the estimation stage, the indexes ${\hat{q}}_v$, ${\hat{r}}_v$, and ${\hat{s}}_v$ are guessed for every block *v* individually. The estimated indexes are encoded to the corresponding vector ${\hat{\mathbf {x}}}_v$ using the same encoding as described in the previous part. To perform the estimation in the next iteration, the encoded vectors ${\hat{\mathbf {x}}}_v$ are subtracted from the input vector ${\mathbf {y}}$ removing the interference from other vectors in the superposition.

The estimation in block *v* starts with computing the inner products between the inversely permuted input vector and all elements in the associative memory (AM):

14 $$\begin{aligned} {\hat{\mathbf {c}}}_v[q,r] = \frac{1}{D} <\rho ^{-r} \left( \Pi _v^{-1}\left( {\hat{\mathbf {y}}_v}\right) \right) , {\mathbf {e}}_q >, \end{aligned}$$

where $\Pi _v^{-1}(.)$ is the inverse permutation of block *v* and $\rho ^{-r}$ the cyclic shift by $(-r)$ elements. The estimated item and rotation indexes are those that maximize the absolute value of the inner product:

15 $$\begin{aligned} {\hat{q}}_v, {\hat{r}}_v&= \mathop {\text {argmax}}\limits _{q=1,...,N \, \, r=1,...,D} \left| {\hat{\mathbf {c}}}_v[q,r]\right| , \end{aligned}$$

and the estimated sign is the sign of the maximizing inner product:

16 $$\begin{aligned} {\hat{s}}_v&= {\mathrm {sign}}({\hat{\mathbf {c}}}_v[ {\hat{q}}_v,{\hat{r}}_v]). \end{aligned}$$

After encoding the estimated indexes to the vectors ${\hat{\mathbf {x}}}_v$, the input vector is cleaned up for the estimation in the next iteration $i+1$:

17 $$\begin{aligned} {\hat{\mathbf {y}}}_v^{(i+1)}&= {\mathbf {y}} - \sum _{j\ne v} {\hat{\mathbf {x}}}_j^{(i)}. \end{aligned}$$

18 $$\begin{aligned}&= {\mathbf {y}} - \left( \sum _{v=1}^V{\hat{\mathbf {x}}}_j^{(i)}\right) +{\hat{\mathbf {x}}}_v^{(i)}. \end{aligned}$$

In the first iteration, all feedback vectors are initialized to zero, i.e., ${\hat{\mathbf {x}}}_v^{(0)}={\mathbf {0}}$. The decoding is repeated until all estimated indexes converge, or until a maximum number of iterations is reached without convergence. Finally, the estimated indexes are mapped to the bit-string ${\hat{\mathbf {u}}}$.

The computations in the proposed unit-feedback decoder are dominated by the AM search depicted in Eq. (14). These AM searches allow for a high degree of parallelism and only require additions and subtractions, thanks to the bipolar representation of the dictionary. Moreover, the search can be efficiently deployed to a computational memory [42], such as phase-change memory, where the inner product is computed in constant time at ${\mathcal {O}}(1)$ in the analog domain leveraging Kirchhoff’s law. When applied to a language classification problem, performing the AM search in the phase-change memory has shown to be over $100\times$ more energy efficient than in an optimized digital implementation [30].

### Experimental results

This section evaluates the BER vs. SNR performance for Integer-HDM and other state-of-the-art (SoA) codes. We assume an AWGN channel with the received signal in the baseband ${\mathbf {y}}$ being modeled as:

19 $$\begin{aligned} {\mathbf {y}} = {\mathbf {x}} + {\mathbf {n}}, \end{aligned}$$

where ${\mathbf {x}}$ is the sent vector containing *V* accumulated vectors, and ${\mathbf {n}}$ is AWGN with ${\mathbf {n}}\sim {\mathcal {N}}({\mathbf {0}},\frac{V}{\mathrm {SNR}}{\mathbf {I}}_D)$ and ${\mathrm {SNR}}$ the signal-to-noise ratio. We define the energy per information bit over noise floor $E_b/N_0 := {\mathrm {SNR}}/2r$.

**Fig. 3:**
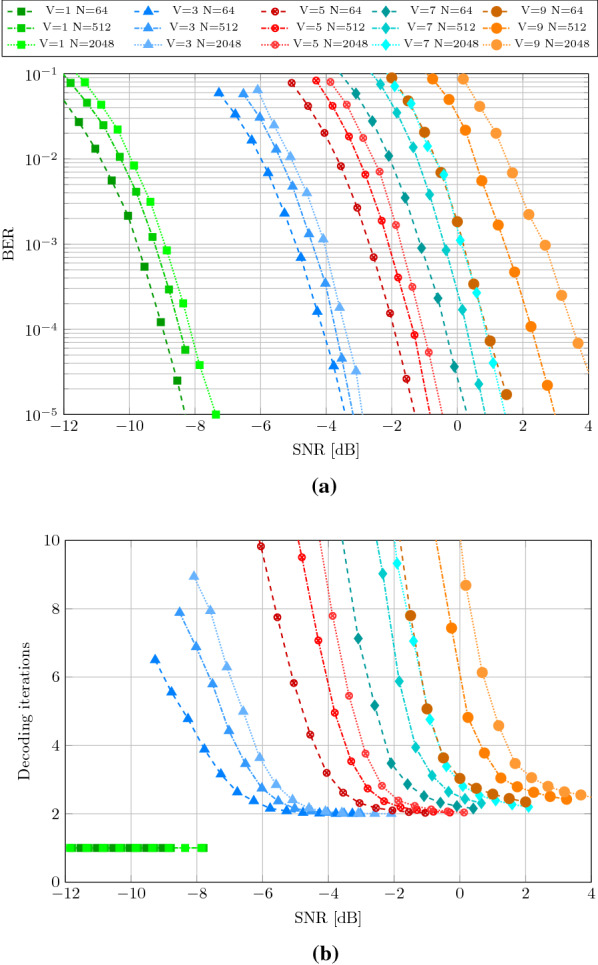
BER (**a**) and number of decoding iterations (**b**) depending on the SNR for Integer-HDM with fixed $D=512$ and varying number of superposed vectors *V* and item memory size *N*

Figure 3a shows the BER vs. SNR behavior of Integer-HDM when varying the number of superposed vectors *V* and the IM size *N* while fixing the dimension to $D=512$. Transmitting a single vector ($V=1$) shows the highest noise resiliency but results in the lowest code throughput ($r=0.031-0.041$ for $N=64-2048$). Integer-HDM allows us to flexibly increase the number of superposed vectors resulting in a linear increase in code throughput; e.g., superposing nine vectors achieves the highest coding rate of $r=0.37$. Transmitting more vectors at the same time reduces the self-induced SIR; hence, a higher SNR is required to achieve the same BER.

The number of decoding iterations of the same code configurations is shown in Fig. 3b. Iterative decoding is not helpful when transmitting only one vector ($V=1$) as no denoising of other superposed vectors is needed; thus, decoding is terminated after the first iteration. Conversely, the number of decoding iterations depends heavily on the number of superposed vectors, the IM size, and the SNR, when superposing more than one vector. However, the number of iterations converges towards two when increasing the SNR. More importantly, a low number of iterations is observed in low BER regimes (where the code is eventually operating); e.g., Integer-HDM in configuration $V=7$ and $N=512$ requires $\approx 0$ dB at $\hbox {BER}=10^{-4}$ and takes only 2.44 decoding iterations at the same SNR.

Next, we compare Integer-HDM to Complex-HDM [33] and a Polar code. Like in Complex-HDM [33], we evaluate the codes in short block lengths ($D=512$) at a throughput of $r=1/4$. Complex-HDM sends vectors with complex-valued elements of block length $D=256$ at a throughput of $r_c=1/2$ bits per *complex* channel use, which is *equivalent* to our setting with $r=1/4$ bits per real channel use and a block length of $D=512$.

The integer codes are configured to $V=7$ and $N=512$, yielding a throughput of $r=0.2598$. A rate 1/4 Polar code at equal block length 512 serves as a second baseline. We use it according to the downlink configuration specified by 3GPP for 5G New Radio (NR) [43]: the information bits are appended by 24 CRC bits and encoded by the Polar encoding with rate-matching. The encoded bits are transmitted with BPSK. For decoding the soft symbols, we use CRC-aided successive cancellation list decoding with list length $L=4$ [44]. As the $L=4$ list decoder utilizes a part of the information in the CRC bits, we count two of these towards the parity bits. We consider the remaining 22 bits as effective information bits for the comparison, as block errors are not detected in the HDM case. As a result, the effective information bits comprised 106 information bits plus 22 CRC information bits for the Polar code.

**Fig. 4:**
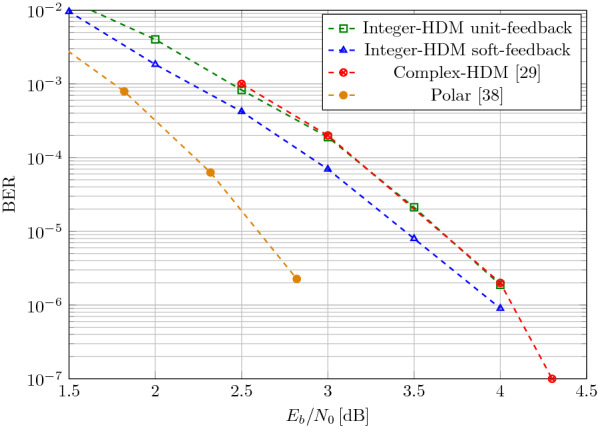
Bit error rate (BER) of considered codes with $k=128$ information bits and $D=512$ real-valued transmission symbols

Figure 4 shows the waterfall diagram of all considered codes. Our proposed Integer-HDM with unit-feedback decoder performs on par with Complex-HDM [33] without needing CRC-aided decoding nor FFT operations. Moreover, it requires fewer decoding iterations than Complex-HDM (2.44 vs. 2.9 @0 dB SNR). The rate 1/4 Polar code outperforms the HD-based codes: it requires  1.2 dB less SNR at BER of $10^{-6}$. However, this comes at the cost of a higher number of decoding operations: Polar codes have shown to require $1.2\times$ more decoding operations than Complex-HDM (336 vs. 280 operations per information bit) [33]. The high decoding complexity has an impact on the overall power consumption of the system that includes encoding, transmission, and decoding [45]. Complex-HDM has already been shown to require fewer decoding operations than Polar codes. We further reduce the number of iterations by lowering the number of decoding iterations and replacing the FFT-based decoding with cheap AM searches, that can be efficiently implemented in the analog domain [30].

## Soft-feedback decoding

**Fig. 5:**
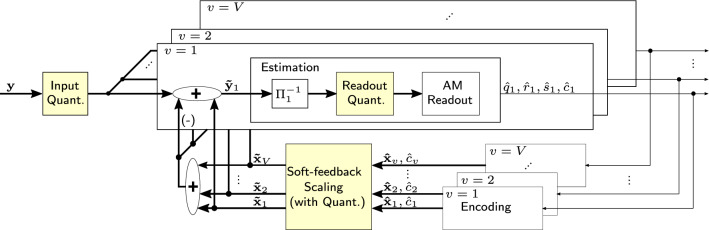
Soft-feedback decoder

This section proposes enhancements to the decoder, introducing a new soft-feedback strategy and quantization schemes for more efficient decoding. Figure 5 depicts the soft-feedback decoding mechanism that scales the currently estimated vector according to the confidence of the previous estimation. Estimations with low confidence are attenuated in the feedback, which results in a damped behavior. We show that the new soft-feedback decoding increases the number of correct vectors retrieved in both the AWGN and noise-free case.

### Soft-feedback decoding

**Fig. 6:**
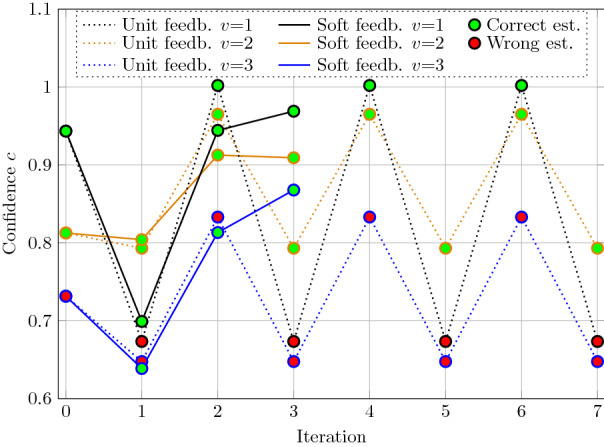
Confidence ${\hat{c}}$ during iterative decoding of $V=3$ vectors using either unit-feedback or soft-feedback. The correct estimations are marked in green and the incorrect in red

The feedback stage reconstructs the estimated vector to remove the noise from the superposition in order to increase the SIR. However, it is not clear in advance how much the past estimations should influence the future ones. The unit-feedback strategy, used both in Complex-HDM and our standard Integer-HDM, weighs all estimations equally with factor one, which can have limitations. For example, if the number of wrong estimations outweighs the correct ones, the feedback decreases the SIR instead of increasing it. Moreover, we observed oscillatory behavior in the unit-feedback decoder, illustrated in Fig. 6.

To this end, we propose a soft-feedback scaling function, which attenuates estimations with low confidence:

20 $$\begin{aligned} {\tilde{\mathbf {x}}}_v = {\mathrm {max}}\left( {\hat{c}}_v,1\right) \cdot {\hat{\mathbf {x}}}_v, \end{aligned}$$

where ${\hat{c}}_v := \left| {\hat{\mathbf {c}}}_v[ {\hat{q}}_v,{\hat{r}}_v])\right|$ is the highest absolute inner product interpreted as the confidence of the previous estimation. As the inner product can exceed one, we limit the feedback scaling to be less or equal to one. The example in Fig. 6 illustrates the soft-feedback scaling’s effectiveness: the oscillations are no longer present, and we converge to the correct solution.

### Quantized Soft-feedback decoding

For most FEC codes, the decoding complexity is significantly higher than the coding complexity. This also holds for our proposed Integer-HDM; therefore, any reduction of the computational requirements for decoding is desirable. We start by quantizing the decoder to fixed-point, where we quantize every value in the decoder to a fixed-point representation with *m* magnitude bits (integer) and *q* fractional bits, denoted as “fixed-point *m*.*q*”. The quantization has the main effect on the input vector ${\mathbf {y}}$ as well as the damped feedback vector ${\tilde{\mathbf {x}}}$. The range of expected values of the input vector depends on the number of added vectors *V*. For example, with $V=3$, we expect values in $\lbrace -3, -1, 1, 3\rbrace$, which can be represented by $m=3$ integer bits. If we reduced the number of integer bits, high values get clipped, which is not desirable in the decoding process. The feedback scaling takes values in [0, 1]; a quantization to $q=1$ fractional bits and arbitrary *m* yields scaling factors in $\lbrace 0, 0.5, 1 \rbrace$.

In addition to the quantization of the general decoder to fixed-point arithmetic, we further reduce the complexity by quantizing the AM search. The dominating operation in the AM search is the inner product between the query vector ${\tilde{\mathbf {y}}}$ and all vectors in the dictionary ${\mathbf {e}}_q \in \lbrace -1,1\rbrace ^D$. We quantize the query vector before the AM search by mapping it to the nearest neighbor from the set of values in the original, noise-free case:

21 $$\begin{aligned} {\mathrm {Q}}({\mathbf {y}}[i],V') = \mathop {\hbox {argmin}}\limits _{l= -V',-V'+2,...,V'-2,V'}||{\mathbf {y}}[i]-l||_2 . \end{aligned}$$

**Fig. 7:**
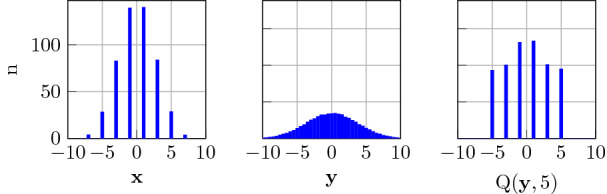
Histogram of encoded vector ${\mathbf {x}}$ with $D=512$ and $V=7$, received vector ${\mathbf {y}}$ at 0 dB SNR, and readout-quantized vector with $V'=5$

Figure 7 shows the histograms of the elements in an encoded vector with dimension $D=512$ and $V=7$. The elements in ${\mathbf {x}}$ take values in $\lbrace -7,-5,...,5,7\rbrace$, whereas values with large amplitude are less probable than small values, which are close to 0. We then add AWGN (0 dB SNR) to the encoded vector, yielding ${\mathbf {y}}$. In the readout-quantization, we map the values to the nearest neighbor of the values in the original, noise-free case. Moreover, we limit the values to $V'$ due to the low probability of values with large amplitudes. In the extreme case, we set $V'=1$, which would reduce the inner product to a Hamming similarity computation. If $V'>1$, the inner product can be computed with integer or binary arithmetic, mapping the values to a Thermometer code.

### MMSE-optimized readout

We consider an alternative AM readout matrix to ${\mathbf {E}}$ determined by minimizing the mean-squared error between the estimated ${\hat{\mathbf {c}}}_v$ and the ground truth vector ${\mathbf {c}}_v$ [14]:

22 $$\begin{aligned} {\hat{\mathbf {c}}}_v = {\mathbf {F}}^T_v \cdot {\mathbf {x}}, \end{aligned}$$

where we assume no sign and rotation encoding for simplicity. The minimum mean square error (MMSE) estimator can be found by solving a linear regression problem, providing a training set of *R* samples with ground truth symbol vectors ${\mathbf {c}}_v$ and their encoded HD superposition ${\mathbf {x}}$. The MMSE readout matrix ${\mathbf {F}}$ can be found with stochastic gradient descent (SGD) minimizing the MSE between ground truth symbol vectors ${\mathbf {c}}_v$ and estimated symbol vectors ${\hat{\mathbf {c}}}_v$ on the training. Note that we neither have to inversely permute the superposition ${\mathbf {x}}$ nor require the knowledge of the underlying dictionary; the readout matrix is only learned based on empirical data. However, a separate readout matrix ${\mathbf {F}}_v$ is needed for every superposed vector, which increases the memory footprint, specifically with large *V*.

The MMSE readout has been shown to increase the number of superposed vectors that can be successfully retrieved with high probability $p_c$ [14], compared to the standard AM search. Consequently, this results in a higher operational capacity of the superposition which is defined as the number of bits/dimension:

23 $$\begin{aligned} {\mathrm {Capacity}}\left( p_{c}\right)&= \frac{V}{D}\left\{ p_{c} {\mathrm {log}}_2 \left( p_{c} N\right) \right. \nonumber \\&\left. + \left( 1-p_{c}\right) {\mathrm {log}}_2 \left( \frac{N}{N-1}\left( 1- p_{c}\right) \right) \right\} . \end{aligned}$$

### Experimental results

We compare our novel soft-feedback decoder in AWGN simulation using both full-precision floating-point and quantized decoder. Moreover, we evaluate the accuracy of the correct retrieval of HD superpositions in the noise-free case using different decoding strategies.

#### Soft-feedback decoding

First, we compare the soft-feedback with the unit-feedback decoder used in Integer-HDM and Complex-HDM, shown in Fig 4. The Integer-HDM code is in the same configuration as in the previous experiment (i.e., $D=512$, $N=512$, and $V=7$). The soft-feedback decoder is able to increase the SNR gain by 0.2 dB compared to the unit-feedback decoder. As a result, Integer-HDM with soft-feedback reduces the SNR gap to the Polar 1/4 code (0.7 dB gap at $\hbox {BER}=10^{-4}$ and 0.8 dB at $\hbox {BER}=10^{-5}$).

#### Quantized Soft-feedback decoding

**Fig. 8:**
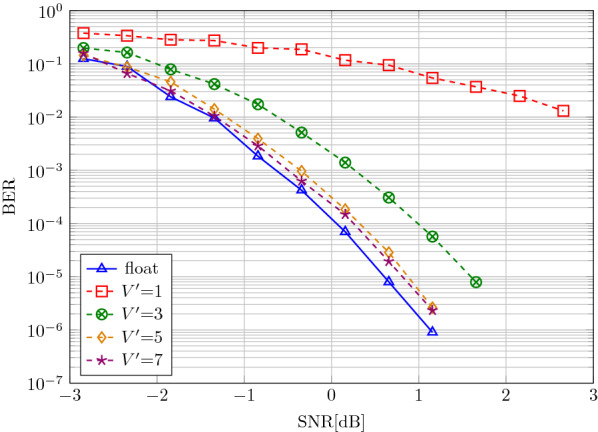
AM readout-quantization 1/4 rate HDM soft-feedback decoder with $D=512$, $N=512$, and $V=7$

We analyze the performance of the soft-feedback decoder when quantizing specific parts of the decoder, described in Sect. 4.2. We start with the quantization of the AM readout, i.e., the values in the query vectors ${\tilde{\mathbf {y}}}$ fed to the AM readout. The results in Fig. 8 illustrate that when quantizing the vector elements to bipolar values (i.e., $\lbrace -1, 1\rbrace$ at $V'=1$), the code performance degrades significantly, compared to the full-precision AM readout. Similar degradation was observed when quantizing the encoded vector ${\mathbf {x}}$ to bipolar values before sending it over the channel. On allowing more levels ($V'=7$), however, the code performance can be re-established.

**Fig. 9:**
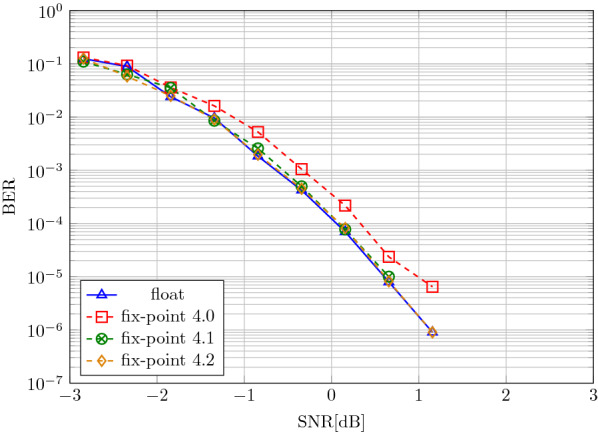
Decoder quantization 1/4 rate HDM soft-feedback decoder with $D=512$, $N=512$, and $V=7$

When quantizing the entire decoding to fixed-point arithmetic (see Fig. 9), one fractional and four integer bits are sufficient to achieve the same performance as the decoder in floating-point. In addition to the desired reduction in decoding complexity, this result also gives valuable insight into the soft-feedback decoder: a feedback scale taking values in $c \in \lbrace 0, 0.5, 1\rbrace$ is sufficient. This yields three options for feedback: take estimation fully into account ($c=1$), ignore it ($c=0$), or partly use it ($c=0.5$).

#### Recall from noise-free superpositions

Finally, we experimentally evaluate the decoding performance of the presented feedback decoder and different readout matrices (standard AM and MMSE) in the noise-free case. We measure the probability of correct retrieval $p_c$ and derive the operational capacity as in (23). For comparison, we use the same configurations as in [14]: we fix the dimension $D=500$ and vary the IM size $N\in \lbrace 5,15,100\rbrace$ and the number of superposed vectors $V\in \lbrace 1,2,...,300\rbrace$. No sign and rotation encoding are used in these experiments.

**Fig. 10:**
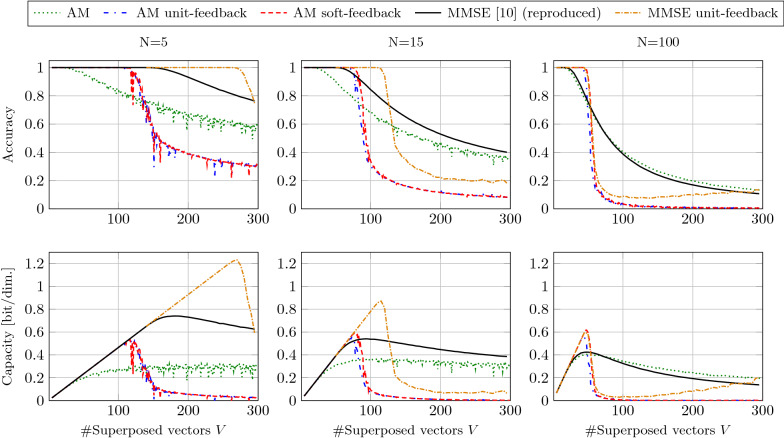
Top: accuracy of correct retrieval of compressed representation of *V* accumulated vectors of dimension $D=500$ with dictionary size of $N=5$, $N=15$, and $N=100$. Bottom: capacity derived from accuracy reported in bits per vector dimension

Figure 10 shows the accuracy and the resulting capacity for the decoder without feedback, with unit-feedback, and soft-feedback. Moreover, we conducted experiments with the MMSE estimator with and without feedback. The MMSE decoder performed similarly with unit and soft-feedback; therefore, we only show unit-feedback results.

Considering the estimator's accuracy without feedback in small IM sizes ($N=5$), the MMSE readout can decode a much larger number of superposed vectors with 100% accuracy, compared to the standard AM readout ($V=134$ vs. $V=12$). However, the advantage of MMSE over AM readout vanishes when increasing the IM sizes ($N=100$).

The feedback decoder significantly increases the number of correctly retrieved vectors in small IM size when using both the MMSE and AM readout ($V=250$ and $V=100$ for AM soft-feedback and MMSE unit-feedback, respectively). Moreover, the soft-feedback further increases the accuracy compared to unit-feedback, especially in larger IM sizes ($N=100$). Generally, the feedback decoder moves the corner point of 100% correct recoveries to larger *V*s; however, the accuracy descent is much steeper compared to non-iterative estimations. The later yet steeper descent of the feedback decoder shows that the denoising is only effective until a certain SIR (i.e., the number of added vectors *V*). If the SIR gets too low, most of the estimations are wrong, and the feedback adds even more interference.

Considering the capacity, MMSE unit-feedback significantly improves the capacity in small dictionary sizes ($N=5$) compared to the current SoA MMSE readout (1.2 vs. 0.7 bits/dimension). This capacity cannot be achieved in larger dictionary sizes. On the contrary, the AM readout with unit or soft-feedback keeps the maximum capacity constant ($\approx 0.6$ bits/dimension), with the soft-feedback achieving slightly higher capacity than the unit-feedback.

## Case study: hybrid near-channel classification and data transmission in EMG-based gesture recognition

**Fig. 11:**
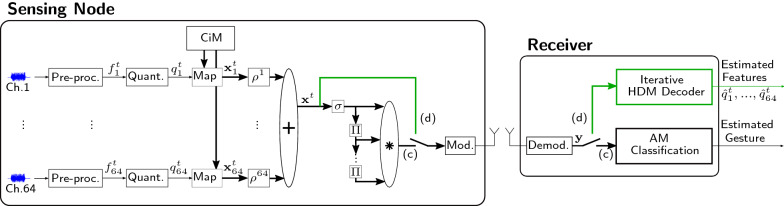
EMG use-case with hybrid modes: a classification mode (c), and a data transmission mode (d). Both modes use the same spatial encoder consisting of pre-processing, quantization to 128 levels, mapping to HD vectors with continuous item memory (CiM), and superposition. In classification mode (c), *n* subsequent vectors are converted to bipolar vectors with $\sigma (.)$, encoded to an n-gram, transmitted over a wireless channel, and classified with the AM search. In data transmission mode (d), the spatial vector is directly transmitted over the channel and decomposed using the iterative HDM decoder

This section extends the application of pure data transmission with a classification task in EMG-based gesture recognition [22], illustrated in Fig. 11. Our hybrid system provides two modes: (1) a classification mode, where the received bipolar vector is used to estimate the gesture using an AM search; (2) a data transmission mode, where the quantized features are reconstructed at the receiver for further analysis. In related work, alternative hybrid approaches compress EMG data using rakeness-based compressed sensing [46] or with a stacked auto encoder [47], before sending the data to the receiver. The received data can be reconstructed or classified using an artificial neural network (ANN). However, these representations are sensitive to noise when used in connection with ANNs [48], while the HD representation in our approach is naturally robust against noise, as we will experimentally show in this section.

### flexEMG dataset

We use the dataset from a study in [22], which contains recordings of three healthy, male subjects. Each subject participated in three sessions recorded on three different days. We only use sessions one and three, which contain a separate training set and test set. The subjects performed four different gestures (fist, raise, lower, open) plus the rest class in ten runs, yielding a total of $10\cdot 5=50$ trials per training and test set. The data were acquired with 64 electrodes, uniformly distributed on a flexible $16 \times 4$ grid of size 29.3 cm $\times 8.2$ cm. Finally, the data were sampled at 1 kS/s and sent to a base-station over BLE.

### Hybrid encoding

#### Classification

We propose a spatiotemporal encoding, which differs from [22] by exclusively using bipolar MAP operations instead of multiplicative mappings. First, the data of every EMG channel is pre-processed the same way, passing it through a digital notch filter with a 60 Hz stopband and a Q-factor of 50, an 8th-order Butterworth band-pass filter (1–200 Hz), an absolute value computation, a moving average filter with 100 taps, and then downsampled by $100\times$, yielding ten samples per second. Moreover, the samples are normalized with the 95% quantile of the training data per channel, which results in features $f_{\mathrm{ch}}^t$ in [0, 1] with high probability (i.e., $p=0.95$ on the training set).

For mapping features to HD vectors, we quantize them to $L=128$ levels and map them to a corresponding value vector stored in a continuous IM (CiM) [23]. The CiM is shared among all channels and is constructed as follows. First, a bipolar seed vector is drawn randomly, which corresponds to level $l=1$ . For level $l=2$, we invert *D*/(2*L*) values at random positions. For the remaining levels, we continue inverting an increasing number of bits until we have inverted *D*/2 elements for level $l=L$, which yields orthogonal vectors for level $l=1$ and $l=L$. This mapping is fully bipolar and more hardware-friendly than the multiplicative mapping used in [22], which relies on multiplicative floating-point operations.

The embedded value vector is circularly permuted, depending on the channel index, and superposed resulting in the compressed representation ${\mathbf {x}}^t$. The encoding is completed by bipolarizing ${\mathbf {x}}^t$ and building a 5-gram out of five consecutive vectors with random permutations ($\Pi$) and binding ($*$). Overall, the encoding achieves a throughput of

24 $$\begin{aligned} r=\frac{64{\mathrm {\,channels}}\cdot 7{\mathrm {\,bits}}\cdot 5{\mathrm {\,gram}}}{D}, \end{aligned}$$

which can, depending on the dimension of the HD vector, result in compression (e.g., $r=4.375$@$D=512$).

The encoded vector is modulated (e.g., with BPSK) and sent to the receiver over a wireless channel. At the receiver, the demodulated signal ${\mathbf {y}}$ is finally classified with an AM search. The AM stores a prototype vector per class. Each prototype is learned by accumulating all encoded vectors of the training samples for each class and finally bipolarizing the vectors. For classification, the query vector ${\mathbf {y}}$ is compared to all prototype vectors using the AM readout. The class with the corresponding best matching prototype is the estimated label [23].

#### Data transmission

The availability of the underlying data, which led to a certain decision or classification, can be helpful in many applications, e.g., allowing interpretability of the model or analysis of the data by a medical specialist. To address this demand, we propose an additional data transmission mode, where the spatially encoded vector ${\mathbf {x}}^t$ is sent to the receiver and decoded with an iterative HDM decoder. This comes with minimal additional requirements at the sensing node, compared to the standard approaches where features are encoded with separate source and channel coding.

In contrast to the quasi-orthogonal IM used for encoding in the previous Sect.  3, the CiM is non-orthogonal, i.e., not every quantization level $q_i$ has an orthogonal vector. This makes the exact decoding of the features difficult; however, the distance preserving CiM mapping reduces the effective error in the reconstruction. For example, an estimation of ${\mathbf {e}}_{q+1}$ instead of ${\mathbf {e}}_{q}$ translates to an error of only 1/L.

### Experimental results

#### Classification

We assess the classification performance in the noise-free, single-node AWGN, and multi-node interference case. The classification accuracy is defined as the ratio between the number of correct estimations and the total number of estimations, given that the classifier makes a new estimation every 100 ms. All models were implemented and tested in MATLAB 2019b.

Table 1 shows the classification accuracy in the noise-free case. A support vector machine (SVM) with linear kernel and cost parameter $C=500$ on pre-processed, flattened features in float-32 precision with dimension 320 (64 channels 5-gram) [49] as well as an HD classifier with multiplicative mapping [22] serve as baselines. Both HD classifiers operate at a dimension of $D=10,000$. The SVM marginally outperforms the HD classifiers by 0.14% and 2%; however, in contrast to the HD classifiers, the SVM does not support online updates of the model, which is crucial for practical deployment of EMG applications [49]. The bipolar feature embedding using the CiM instead of the float-based multiplicative mapping in the HD classification yields only a small accuracy degradation (95.99% vs. 94.13%).

**Table 1:** Classification accuracy (%) on 5-class EMG-based gesture recognition task using 64-channel flexEMG data [22]

Classifier Representation		SVM^a^ [49] Float	HD^a^ [22] Float	HD (ours) Bipolar
Subject	Session			
1	1	98.13	99.60	97.20
	3	100.00	99.20	98.20
2	1	99.53	98.33	98.53
	3	96.47	97.53	96.07
3	1	99.60	90.40	92.27
	3	83.07	90.87	82.53
Average		96.13	95.99	94.13

We compare a linear SVM, an HD classifier with multiplicative embedding, and our HD classifier with bipolar CiM embedding. Both HD classifiers operate at dimension $D=10\,000$

^a^Reproduced

**Fig. 12:**
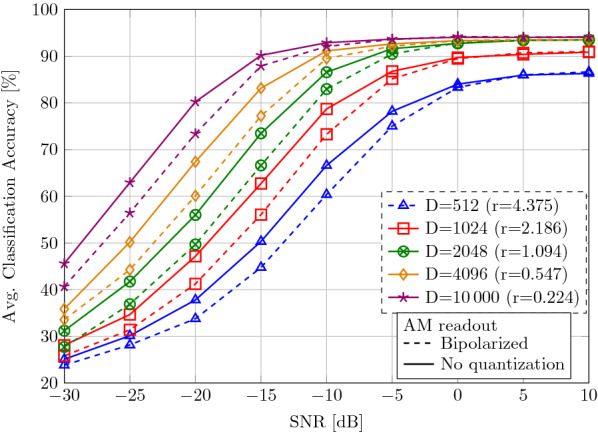
Classification accuracy (%) in 5-class gesture recognition on 64-channel EMG data. The transmitted HD vector is interfered with AWGN

Next, we evaluate the classification accuracy when the query vector was exposed to noise:

25 $$\begin{aligned} {\mathbf {y}} = {\mathbf {x}} + {\mathbf {n}}, \end{aligned}$$

where ${\mathbf {x}} \in \lbrace -1,1\rbrace ^D$ is the encoded vector and ${\mathbf {n}}\sim N({\mathbf {0}},\frac{1}{\mathrm {SNR}}{\mathbf {I}}_D)$ AWGN. Figure 12 shows the average classification accuracy for different vector dimensions, depending on the SNR. In the high SNR regime (SNR = 10 dB), a reduction in the dimension results in slight accuracy degradation (e.g., 93.91%@$D=8192$ vs. 86.32%@$D=512$). When decreasing the SNR, we see a graceful accuracy degradation with superior performance when using higher dimension: at $D=4096$, the absolute accuracy loss compared to the noise-free case is less than 4% in low SNR until $-10$ dB SNR (91.16% vs. 94.13%).

As an additional experiment, we bipolarize the query vector ${\mathbf {y}}$ before the AM search, shown in dashed lines. This allows a more efficient AM search only requiring Hamming distance computation; however, it results in lower classification accuracies in the low SNR regime (SNR < 0 dB).

**Fig. 13:**
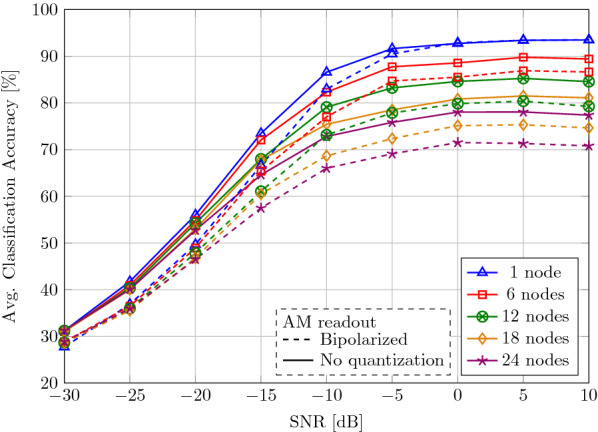
Classification accuracy (%) in 5-class gesture recognition on 64-channel EMG at $D=2048$. The transmitted HD vector is interfered with other nodes

Furthermore, we demonstrate the robustness of our distributed representations in the presence of interference from unrelated nodes as well as AWGN, shown in Fig. 13. The nodes operate at ${D}=2048$ where the effective throughput is $r=1.094$; hence, the encoding does not add any redundancy. The HD representation exhibits robustness against the interference: when interfering with up to 6 nodes at large SNR (10 dB), the classification accuracy drops by only 4.07% (93.50% vs. 89.43%). Moreover, a graceful accuracy degradation is observed at low SNR of $-5$ dB and 6 interfering nodes, where an accuracy of 87.75% is maintained.

#### Reconstruction of features

Finally, we reconstruct the encoded features with the soft-feedback decoder in the presence of AWGN. We measure the mean-squared error (MSE) between reconstructed and original features during active gesture intervals of all subjects in sessions 1 and 3. The time between trials is not considered for reconstruction. Also, the encoded vector is exposed to AWGN.

**Fig. 14:**
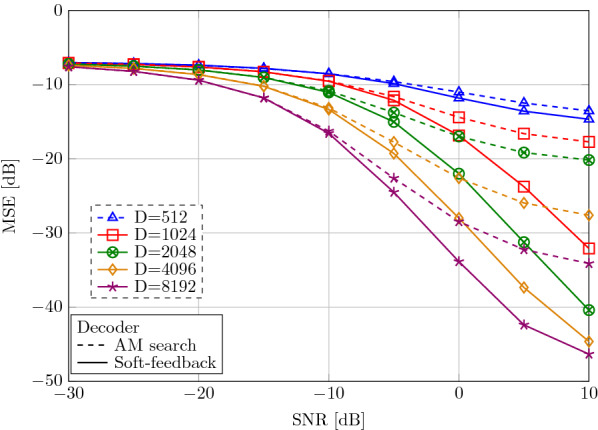
Mean-squared error (MSE) for reconstructing features from spatially encoded vector ${\mathbf {x}}$ decoded with soft-feedback or with AM readout without feedback decoder

Figure 14 shows the MSE depending on the SNR using either the soft-feedback decoder or the AM search without feedback. Akin to previous classification results, higher dimensional representations show higher noise resiliency yielding a lower MSE. Moreover, the soft-feedback further improves the retrieval of the features with up to 10 dB MSE reduction compared to AM readout without feedback. As a result, the soft-feedback decoder allows the vector dimension to be reduced while still ensuring lower MSE: at 10 dB SNR, soft-feedback at dimension $D=2048$–8192 achieves lower MSE than AM readout in all considered dimensions $D\le 8192$. At dimension $D=2048$, the soft-feedback decoder achieves a maximal reconstruction gain of 20 dB MSE at 10 dB SNR compared to AM readout without feedback.

**Fig. 15:**
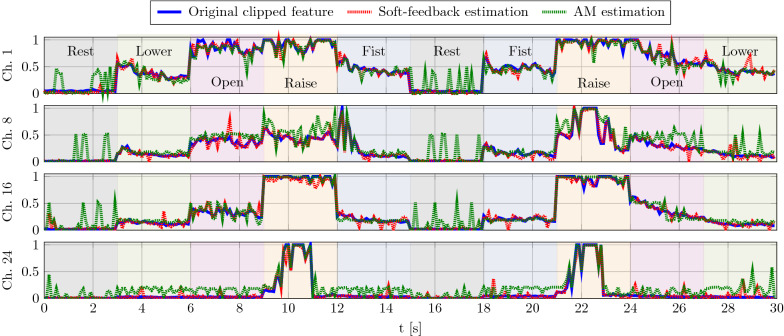
Original and reconstructed EMG features of subject one with $D=1024$ and 5 dB SNR. Ten gesture trials of 3 s length are concatenated; the break between trials was removed. Displayed, reconstructed signal has $-25.1$ dB MSE with soft-feedback decoder and − 18.2 dB MSE with AM readout

For illustration, Fig. 15 depicts the original features of subject 1 in the training session of the first session, the reconstructed features with soft-feedback decoder, and the reconstructed features with the AM readout without feedback. The reconstructed features from the AM readout without feedback shows many faulty estimations that do not follow the ground truth, being particularly visible as peaks during the rest state. In contrast, the soft-feedback decoder’s estimation follows the ground truth more accurately.

## Conclusion

This paper investigates the use of robust and distributed HD representations in wireless communication and classification. We propose a novel encoding, called Integer-HDM, that generates integer-valued vectors based on bipolar seed vectors, cyclic shift encoding, sign modulation, and superposition. A new soft-feedback decoder successfully decomposes the vectors, improving the decoding performance in both noise-free and AWGN scenarios. Achieving a similar SNR gain as complex HDM [33], the proposed Integer-HDM does not require FFT operations and can be quantized to low-resolution fixed-point arithmetic. In a classification use-case, an EMG-based hand gesture recognition demonstrates the robustness of HD representations against AWGN and other interfering sensing nodes; and thus, the same spatial encoding can be used for classification as well as reconstruction of the underlying features. Further investigations can be made into the decoding of bipolarized superpositions, and N-gram encoded vectors, e.g., using resonator networks [50, 51].

## Data Availability

The flexEMG dataset analyzed during the current study is available under https://github.com/a-moin/flexemg [22].
